# Research protocol of the NeedYD-study (Needs in Young onset Dementia): a prospective cohort study on the needs and course of early onset dementia

**DOI:** 10.1186/1471-2318-10-13

**Published:** 2010-03-12

**Authors:** Deliane van Vliet, Christian Bakker, Raymond TCM Koopmans, Myrra JFJ Vernooij-Dassen, Frans RJ Verhey, Marjolein E de Vugt

**Affiliations:** 1 School for Mental Health and Neuroscience, Alzheimer Center Limburg, Maastricht University Medical Center, Maastricht, the Netherlands; 2 Florence, Mariahoeve, Center for Specialized Care in Early Onset Dementia, Den Haag, the Netherlands; 3 Department of Primary and Community Care: Center for Family Medicine, Geriatric Care and Public Health, Radboud University Nijmegen, Medical Centre, Nijmegen, the Netherlands; 4 Scientific Institute for Quality of Healthcare/Alzheimer Center Nijmegen, Radboud University Nijmegen, Medical Centre, Nijmegen, The Netherlands; 5 Kalorama Foundation, Beek-Ubbergen, the Netherlands

## Abstract

**Background:**

Early onset dementia has serious consequences for patients and their family members. Although there has been growing attention for this patient group, health care services are still mainly targeted at the elderly. Specific knowledge of the needs of early onset dementia patients and their families is limited but necessary for the development of adequate health care services and specific guidelines. This research project is mainly targeted at delineating the course of early onset dementia, the functional characteristics and needs of early onset dementia patients and their caregivers, the risk factors for institutionalization and the interaction with the caring environment.

**Methods/Design:**

The NeedYD-study (Needs in Young Onset Dementia) is a longitudinal observational study investigating early onset dementia patients and their caregivers (n = 217). Assessments are performed every six months over two years and consist of interviews and questionnaires with patients and caregivers. The main outcomes are (1) the needs of patients and caregivers, as measured by the Camberwell Assessment of Needs for the Elderly (CANE) and (2) neuropsychiatric symptoms, as measured by the NeuroPsychiatric Inventory (NPI). Qualitative analyses will be performed in order to obtain more in-depth information on the experiences of EOD patients and their family members. The results of this study will be compared with comparable data on late onset dementia from a historical cohort.

**Discussion:**

The study protocol of the NeedYD-study is presented here. To our knowledge, this study is the first prospective cohort study in this research area. Although some limitations exist, these do not outweigh the strong points of this study design.

## Background

Dementia is often regarded as a disease of old age. However, there is also a group in which the symptoms of the disease develop before the age of 65. Prevalence rates of early onset dementia (EOD) have been reported to range between 54 and 260 cases per 100,000 in the 30-64 age group [1-3].

EOD is recognized as an important psychosocial and medical health problem with serious consequences for patients and their families [4,5]. EOD is more difficult to recognize than late onset dementia (LOD) in the early stages of the disease because of the lower prevalence rate, the wider range of etiologies [6,7] and the use of other mental health services (e.g., community mental health teams). These factors cause an important delay before an accurate diagnosis can be established, commonly resulting in feelings of insecurity and frustration for both patients and their families [8]. A proper diagnosis is an important prerequisite for receiving adequate (in)formal support and health care services.

EOD also may have a different clinical manifestation than LOD due to the relatively high prevalence rate of frontotemporal dementia (FTD), in which problem behavior is more prevalent as the presenting sign of probable dementia [6,7]. Recent research on the impact of problem behavior on caregivers and vice versa shows that these specific aspects of the dementia, more so than cognitive and functional changes, have severe consequences for patients and their family members [9]. Problem behavior is the most important risk factor for caregiver burden and is a strong predictor of institutionalization [10-12] but is also an important starting point for interventions [13]. Recent studies have shown that psychological factors, such as disease awareness [14], and environmental factors, such as caregiver management strategies [15], influence the development and persistence of problem behavior in LOD. Similar studies on EOD have not yet been conducted.

Furthermore, EOD patients are in a life phase in which they often have an active role in society and often have young children. The loss of roles and responsibilities is, therefore, greater than in older people. They also have to deal with specific issues such as marital problems, family conflict, (un)employment and financial issues [5]. Furthermore, many EOD patients of the post-war baby boom generation grew up in a society that is very different from that of the older generation. The needs of EOD patients may, therefore, be different and demand a different approach than in LOD.

Despite these differences, the availability of specialized healthcare services is still limited in most countries, forcing EOD patients and their family members to use services designed for the elderly. In the Netherlands, specialized services are available, but their geographic distribution is limited, as is the range of services offered. Furthermore, specific knowledge on the characteristics and needs of EOD patients and their families is lacking but prerequisite for the development of suitable health care services. Adequate diagnostics, (in)formal support and services like support groups, day care facilities or respite care may help patients and their families cope with the situation and may even postpone institutionalization. This multidisciplinary research project focuses on the course of EOD, the functional characteristics of EOD patients, the needs of EOD patients and their caregivers, the risk factors for institutionalization and the interaction with the caring environment. We expect that the study will yield important data that can be used to design specific guidelines and improve the development of health care services for EOD patients and their families.

## Aim and research questions

NeedYD is a prospective cohort study with the following primary objectives: (1) to investigate the (un)met needs of EOD patients and their family members during different phases in the course of the disease (e.g., the diagnostic phase and the phase in which (specialized) day care is provided) and (2) to investigate the course of neuropsychiatric symptoms and possible risk factors (i.e., comorbidity, age, communication problems, biological factors, disease awareness, interaction with the environment).

The secondary objectives are:

• To gain insight into the course of other functional domains in EOD (cognition, activities of daily living);

• To explore the experiences and feelings of patients and their caregivers during the diagnostic period;

• To investigate the impact of the diagnosis of dementia on EOD patients and their family members;

• To study the course of the functioning of the caregivers of EOD patients and the problems they (and possibly other family members) experience;

• To identify factors that influence the use of respite care and determine institutionalization;

• To explore to what extent stigma and taboo concerning dementia interfere with adequate communication within the family;

• To compare these findings with findings of studies on LOD.

## Methods/Design

### Design

The NeedYD-study is a prospective cohort study with a follow-up of two years in which a group of EOD patients and their families are assessed at six month intervals. The study design is similar to that of the MAAstricht Study of BEhavior in Dementia (MAASBED) and the WAAL BEhavior in Dementia (WAALBED) study [16-19] conducted in the Netherlands.

### Subjects

The study population consists of dyads of patients with EOD and their caregivers. Patients with onset of disease symptoms before the age of 65 are included in the study (age at inclusion could be later than 65). Diagnoses of dementia subtype are made according to regular criteria [20-25]. Patients are recruited through the memory clinics of the three Alzheimer's centers in the Netherlands located in Amsterdam, Nijmegen and Maastricht, the memory clinics of general hospitals and through other mental health services in the south of the Netherlands as well as through specialized day care facilities that are affiliated with the Dutch National EOD Taskforce. Thus, a group of patients without day care or receiving non-specialized day care, as well as a group of patients receiving specialized day care, are included in the study. For some of the research questions, these groups will be compared. The exclusion criteria are: (1) dementia caused by HIV, traumatic brain injury, Down's syndrome, Huntington's chorea or alcohol-related dementia, (2) lack of a reliable informant or (3) lack of informed consent of the participant. Furthermore, children of EOD patients who are living at home and are older than 14 years of age at the time of the baseline assessments are recruited through their parents.

### Measures

#### Primary outcome measures

(Un)met needs are assessed with the Dutch version of the Camberwell Assessment of Needs in the Elderly (CANE) [26]. This assessment is a semi-structured interview consisting of 24 domains that cover social, physical, psychological and environmental needs. The interview starts with an open question concerning a specific domain, followed by questions regarding help and (in)formal support the patient receives in that particular domain, as well as the amount of help and support that is needed. These items are scored on a three point scale ranging from little (1) to a lot of help (3). Satisfaction with the amount and quality of the help and support received is also assessed. The answers are used to determine whether or not the participant experiences a need and whether or not this need is met. The experienced needs of patients are based on patient and proxy (primary caregiver) interviews. The need for information and the psychological needs of the caregiver are also assessed by means of the CANE. Reliability and validity were found to be adequate [26].

Neuropsychiatric symptoms in the patient and related caregiver burden are assessed with the Dutch version of the Neuropsychiatric Inventory (NPI) [27]. The NPI is a structured interview with the primary caregiver and, when available, a health care professional. After institutionalization, the nursing home version of the NPI (NPI-NH) is used [28]. Ten neuropsychiatric and two neuro-vegetative symptoms are assessed: delusions, hallucinations, agitation/aggression, dysphoria, anxiety, euphoria, apathy, disinhibition, irritability/lability, aberrant motor behavior, night-time behavior disturbances and appetite/eating abnormalities. Screening questions are asked to determine whether behavioral changes are present. In the case of a positive answer, further questions are asked and the severity and frequency of the behavioral disturbances are determined. The Dutch version of the NPI has high inter-rater agreement and is found to be a valid rating scale for measuring a wide range of behavioral and psychological symptoms of dementia [29]. Furthermore, the experience of caregiver distress due to these neuropsychiatric symptoms is determined according to the six point NPI caregiver distress scale (NPI-D) ranging from no distress (0) to extreme distress (5) [30]. The NPI-D provides a reliable and valid measure of subjective caregiver distress in relation to the neuropsychiatric symptoms measured by the NPI.

For an overview of all measurements see Table 1.

**Table 1:** Flowchart of measures used during the assessments

Outcome measure	Operationalization (Type of instrument)	Time of assessment					
***Patient***		**S**	**B**	**F1**	**F2**	**F3**	**F4**
*Primary outcomes*							
Needs	CANE [26] (SSI)		P/C	P/C	P/C	P/C	P/C
Frequency and severity neuropsychiatric symptoms	NPI [27] NPI-NH [28] (SI)		C/N	C/N	C/N	C/N	C/N
*Secondary outcomes*							
Severity of dementia	GDS [31] (RS)		C/P	C/P	C/P	C/P	C/P
Depressive symptoms	CSDD [41] (SI)		C	C	C	C	C
Cognitive functioning	MMSE [33] (CT)		P	P	P	P	P
Cognitive functioning	SIB [34] If MMSE <15 (CT)		P	P	P	P	P
Executive functioning	FAB [36] (CT)		P		P		P
ADL disabilities	IDDD [32] (Q)		C		C		C
Disease awareness	GRAD [37,38] (SSI)		C/P	C/P	C/P	C/P	C/P
Amount of formal and informal care	RUD-lite [42] (SI)		C	C	C	C	C
Quality of life	QoL-AD [40] (SI/Q)		C/P	C/P	C/P	C/P	C/P
*Additional variables*							
Inclusion/exclusion criteria		R/P/C					
Informed Consent		P/C					
Demographic data	Age, gender, education level, marital status, employment		P/C				
Diagnosis	First complaints, date of diagnosis, physician that gave diagnosis		P/C				
Life events	Disease, institutionalization, conflict, divorce, other		P/C	P/C	P/C	P/C	P/C
Medical record investigation	Current diagnosis, possible prior diagnoses, examinations that lead to diagnosis, medical history		R				
Treatment and other information	Physical complaints, current treatment/use of formal care, medical history, substance use, dementia/genetic diseases in family		P/C	P/C	P/C	P/C	P/C
***Caregiver***							
*Primary outcomes*							
Needs	CANE [26] (SSI)		C	C	C	C	C
Experienced burden as a result of behavioral disturbances	NPI [27] (SI)		C/N	C/N	C/N	C/N	C/N
Needs and experiences	(SSI)		C		C		C
Sense of competence	SSCQ [43] (SI)		C	C	C	C	C
*Secondary outcomes*							
Depressive symptoms	MADRS [44]. (SI)		C		C		C
Psychological and somatic complaints	SCL-90 [45] (Q)		C	C	C	C	C
Coping strategies	UCL (Schreurs, Willige et al. 1988) (Q)		C				
Quality of life	RAND-36 [48] (Q)		C		C		C
Quality of the marital relationship	Four items of the University of Southern California Longitudinal Study of Three-Generation Families measures of positive affect [50]. (Q)		C	C	C	C	C
Emotional instability	Subscale neuroticism of NEO-FF-I [52] (Q)		C				
Caregiver management strategy	Caregiver management strategy [15] (SI)		C	C	C	C	C
*Additional variables*							
In/exclusion criteria		C					
Informed consent		C					
Demographic data	Age, gender, education level, marital status, employment		C				
Information on informal care	hours giving care, contact hours, other informal caregivers		C	C	C	C	C
Information on employment	Hours working, date stopped working		C	C	C	C	C
***Children***							
Needs and experiences	(SSI)		Ch				
Demographic data	Age, gender, education level, employment, living situation		Ch				
Data on informal care	Hours spent care giving, contact hours with demented parent		Ch				

S = Screening, B = Baseline, F1 = Follow up measurement 1, F2 = Follow up measurement 2, F3 = Follow up measurement 3, F4 = Follow up measurement, SSI = Semi Structured Interview, SI = Structured Interview, CT = Cognitive Test, Q = Questionnaire, RS = Rating Scale, C = informant is caregiver, P = Informant is patient, N = Informant is health care professional from nursing home, Ch = informant is child

#### Secondary outcome measures for the patient

The Global Deterioration Scale (GDS) is administered to assess the severity of the dementia. The GDS is a widely used instrument which has been validated against behavioral, neuro-anatomic and neurophysiologic measures, for which significant correlations have been found [31]. The Interview for Deterioration in Daily Living in Dementia (IDDD) is used to assess the activities of daily living. The internal consistency of this scale is high (Cronbach's alpha 0.94) [32]. Cognitive functioning is measured using the Mini Mental State Examination (MMSE), which is a reliable and valid test of cognitive function [33]. When the MMSE score is below 15, the Short Severe Impairment Battery (s-SIB) is administered, which has been found to be a reliable and valid test of cognitive function in moderate to severe dementia patients [34,35]. Furthermore, executive functioning is assessed using the Frontal Assessment Battery (FAB). The FAB has good inter-rater reliability, internal consistency and discriminant validity [36]. The Guidelines for the Rating of Awareness of Deficits (GRAD) [37,38] are administered in order to investigate disease awareness. This instrument has substantial inter-rater reliability [39]. The Quality of Life-Alzheimer's Disease scale (QoL-AD), which has good content, criterion and construct validity and excellent inter-rater reliability and internal consistency [40], is used to assess the quality of life of the patient, as perceived by the patient and his caregiver. The Cornell Scale for Depression in Dementia (CSDD) [41] is administered to identify depressive symptoms in the patient. This scale has adequate inter-rater reliability, internal consistency and sensitivity. The amount of formal care the patient receives and the time the caregiver spends caring for the patient are obtained using the Resource Utilization Scale (RUD-Lite), which covers 95% of the resource use, the complete RUD covers [42]. Therefore, it is a good alternative for the complete RUD when the assessment battery is large.

#### Secondary outcome measures for the caregiver

The Short Sense of Competence Questionnaire (SSCQ) [43] is administered to assess caregiver's feelings of being capable to care for a demented individual. The SSCQ was reported to have satisfactory reliability and validity [43]. Depressive symptoms are measured by the Montgomery Asberg Depression Rating Scale (MADRS), which has adequate inter-rater reliability and exhibits construct and concurrent validity [44]. Psychological and physical complaints are measured with the Symptom Checklist 90 (SCL-90). Reliability and construct validity of the SCL-90 are satisfactory [45]. Emotional instability is assessed with the neuroticism subscale of the Dutch version of the NEO-Five Factor Inventory (NEO-FF-I). Internal consistency and test-retest reliability are high for this scale, as is the construct validity [46]. Coping strategies are assessed by means of the Utrechtse Copinglijst (UCL). The reliability of this scale is reasonable and the validity has been found to be sufficient despite some inconsistencies in the literature [47]. General health is measured with the Dutch translation of the RAND-36 [48]. The Dutch version of the RAND-36 appears to be a reliable, valid and sensitive measure for general health [49]. In addition, the quality of the marital relationship and the changes that have occurred since the onset of the disease are measured by four items of the University of Southern California Longitudinal Study of Three-Generation Families measures of positive affect. Cronbach's alpha for this scale is 0.85 [50]. The caregiver management strategy is assessed by means of questions reflecting three caregiver strategies: a caring, supporting or non-adapting strategy [15]. This scale has not yet been validated. Furthermore, a semi-structured interview is administered to the caregiver and, when applicable, to children living at home. The interview includes topics concerning the period prior to diagnosis, the impact of the diagnosis, changes in the interpersonal relationships within the family, the communication within the family about the disease, the problems experienced by the patient and family members, experiences and beliefs concerning (in)formal support and health care services, transitions in care (e.g., day care, institutionalization) and future perspectives.

#### Additional data

By means of a structured interview and examining the patients file information, medical and demographical information of the patient are obtained. For a full description of these data, see Table 1.

### Procedures

EOD patients and their caregivers receive five assessments at six-month intervals (B, F1, F2, F3, F4; Table 1). Before inclusion in the study, (S) information on in/exclusion criteria is collected and informed consent is obtained. Patients who are not able to sign informed consent are asked to give oral consent and their legal representative has to give written consent that the patient can participate. Children living at home who are older than age 14 are asked at baseline to participate in a semi-structured interview. Children aged between 15 and 18 years, as well as their legal representatives, both have to sign informed consent. When individuals do not agree to participate, the reason plus age, gender and diagnosis of the patients are registered.

When participants, after inclusion in the study, do not wish to participate in one of the assessments, caregivers are asked to participate in an interview by telephone, so the CANE, NPI and Sense of Competence questionnaire can be administered and to fill out all of the questionnaires required for that assessment. If this is not possible, the researcher asks them to answer several questions about their own and the patients' functioning and about the use of formal care. If caregivers refuse this as well, the reason for refusal is asked. When a patient has died, data on the use of (in)formal care and the needs of caregivers before and after the patient died is collected from the caregiver as well as the date and cause of death.

### Ethical considerations

The study protocol is approved by the Medical Ethics Committee of the University Medical Center Maastricht. The local ethics committees of the participating institutions have also given consent. The research project is performed according to the principles of the Declaration of Helsinki (version January 2004; http://www.wma.net) and in agreement with the law regarding medical-scientific research in humans (WMO). An independent physician is assigned to the study. Participants are informed about the possibility of contacting him for further questions about the study.

### Sample size

Based on a power calculation (two groups: diagnostic phase and the phase of specialized day care; ANOVA) with an alpha of 0.05, a power of 0.85 and an expected effect size of 0.25, 128 EOD patients are required to participate in the study. With an expected loss to follow-up of 37% in a two year follow-up period based on data of the MAAstricht Study of BEhavior in Dementia (MAASBED) study [15], 200 patients need to be included.

### Data analysis

Data entry is performed twice to safeguard data integrity. Statistical analyses will be performed using the Statistical Package for Social Sciences, version 17. Descriptive statistics will be used to describe characteristics of patients and caregivers, i.e., age, sex, distribution of diagnoses, etc. Both quantitative and qualitative data will be used in the analyses.

Diagnosis matched patients with LOD from a historical cohort (MAASBED study) will be used to make a comparison with EOD. Baseline differences between groups will be analyzed to investigate the comparability of the groups. Depending on the research question and which variables will be analyzed, parametric or non-parametric analyses will be performed. Comparisons between groups will be made with independent samples T-tests or AN(C)OVAs for continuous and normally distributed variables and Pearson's Chi square test, Fisher's exact or Mann-Whitney U tests for categorical and non-normally distributed variables. Comparisons between the memory clinic and day care groups, the EOD group and the LOD group and within groups across measurements will be performed using linear mixed models analyses. A survival analysis will be performed to study predictors of institutionalization. If participants withdraw from the study, they will not be excluded. The data collected can still be analyzed, because of the use of linear mixed models. However, the characteristics of the dropouts and losses to follow-up will be described and taken into account.

Qualitative data will be analyzed using the method of constant comparative analysis [51]. These qualitative analyses will be performed in order to obtain a more in-depth, complex view and understanding of the experiences of EOD patients and their family members. The interviews that are held with the caregivers will be fully transcribed and first read by one researcher. They will then be read a second time to develop codes that will eventually be grouped into categories. Categories will then be grouped into themes. Another researcher will independently apply the same procedure. The analyses will be performed using Atlas.ti.

## Discussion

The current paper presents the study protocol of a prospective cohort study: the NeedYD-study. This project mainly focuses on the course of EOD, the functional characteristics of EOD patients, the needs of EOD patients and their caregivers, the risk factors for institutionalization and the interaction with the caring environment.

To our knowledge, this is the first study that addresses these issues longitudinally in a large cohort. It will contribute widely to our knowledge about the course of EOD, the caring process and the needs of the patient and caregiver as they develop during the course of the disease. A longitudinal design is necessary to examine the predictive value of study variables in observational data. Despite the many positive aspects of this design, there are some limitations.

Sample bias could be a factor in our study. Although patients are recruited through a large range of different institutions, which is likely to be representative of the Dutch population, the group that gives consent may be different from the group that refuses to participate. Furthermore, selective attrition due to early death is inherently associated with the current study.

In addition, the data from the present study and the historical cohort of the LOD sample (MaasBED study) are different in several ways, as the participants are not matched. The LOD patients were mostly seen right after receiving the diagnosis, whereas in the EOD group patients in different stages are included. The EOD group is, therefore, probably more heterogeneous in terms of disease severity, cognitive functioning and ADL disability. Furthermore, the dementia of the EOD patients is possibly more severe because establishing a diagnosis in EOD often takes longer than in LOD. However, these factors as well as other possible confounders are collected in order to take these into account during the statistical analyses.

Furthermore, one may argue that the proxy ratings we use for several patient characteristics are not as reliable as patient ratings. However, in this patient group such ratings are inevitable since dementia patients gradually become cognitively impaired and may suffer from a lack of awareness. Therefore, proxy ratings are preferred to keep the informant during follow-up reliable and constant.

In conclusion, the strong points of this study outweigh its few limitations as long as they are dealt with properly.

## Competing interests

The authors declare that they have no competing interests.

## Authors' contributions

The presented study was designed by FV, RK and MdV. DvV wrote the article and CB assisted with writing the article. FV, RK, MV and MdV critically reviewed the article. All authors read and approved the final manuscript.

## Pre-publication history

The pre-publication history for this paper can be accessed here:

http://www.biomedcentral.com/1471-2318/10/13/prepub
